# The role of small RNAs in resistant melon cultivar against *Phelipanche aegyptiaca* parasitization

**DOI:** 10.3389/fmicb.2024.1408926

**Published:** 2024-05-07

**Authors:** Jian-Cai Mao, Miao Yan, Jun-Hua Li, Jun-Yan Yang, Hao-Jie Wang

**Affiliations:** Hami Melon Research Center, Xinjiang Academy of Agricultural Sciences, Urumqi, China

**Keywords:** trans-kingdom RNAi, melon, broomrape, small RNA sequencing, degradome sequencing

## Abstract

Bidirectional trans-kingdom RNA silencing, a pivotal factor in plant-pathogen interactions, remains less explored in plant host-parasite dynamics. Here, using small RNA sequencing in melon root systems, we investigated microRNA (miRNA) expression variation in resistant and susceptible cultivars pre-and post-infection by the parasitic plant, broomrape. This approach revealed 979 known miRNAs and 110 novel miRNAs across 110 families. When comparing susceptible (F0) and resistant (R0) melon lines with broomrape infection (F25 and R25), 39 significantly differentially expressed miRNAs were observed in F25 vs. F0, 35 in R25 vs. R0, and 5 in R25 vs. F25. Notably, two miRNAs consistently exhibited differential expression across all comparisons, targeting genes linked to plant disease resistance. This suggests their pivotal role in melon’s defense against broomrape. The target genes of these miRNAs were confirmed via degradome sequencing and validated by qRT-PCR, ensuring reliable sequencing outcomes. GO and KEGG analyses shed light on the molecular functions and pathways of these differential miRNAs. Furthermore, our study unveiled four trans-kingdom miRNAs, forming a foundation for exploring melon’s resistance to broomrape.

## 1 Introduction

Broomrape (*Phelipanche aegyptiaca* Pers) is a parasitic plant known for inflicting significant economic losses in melon production. This holoparasite attaches itself to the root of the melon plants and establishes a connection with the vascular tissue to absorb nutrients. This parasitic interaction profoundly impairs host development and substantially diminishes melon yields (Bao et al., 2017). The attachment between broomrape and its host plant is facilitated through specialized structures called haustorium, which enable bidirectional movement of photosynthates and other essential macromolecules between the host and parasite, including microRNAs (miRNAs) (Bao et al., 2017). miRNAs, a class of endogenous single-stranded non-coding RNAs spanning 20–40 nt, are widespread in plants (Nizampatnam et al., 2015; Song et al., 2019; Cui et al., 2020). They play an important regulatory role in plant growth, development, hormone signal transduction, and stress response. Remarkably, miRNAs can even exert regulatory influence over gene expression in different species, highlighting their capacity for cross-species regulation (Guo et al., 2017, 2021). In addition, some miRNAs exhibit the ability to traverse between host and parasite plants, resulting in the reverse silencing of target genes, leading to the phenomenon of “trans-kingdom RNA silencing.” This intriguing process involves the transfer of miRNAs and subsequent reverse silencing of target genes, thus influencing gene expression across the plant-parasite interface (Zhang et al., 2016a,b).

The phenomenon of trans-kingdom RNA silencing was first discovered in the interaction between plants and fungi. Notably, *Botrytis cinerea* demonstrated the ability to transfer a repertoire of small RNAs into plant cells, thereby triggering the silencing of immune response genes across diverse hosts, including *Arabidopsis* and tomato (Weiberg et al., 2013). Subsequent investigations unveiled the mechanism by which pathogenic small RNAs, interfacing with *Arabidopsis* AGO1 and harnessing the host RNAi machinery, silence pivotal host genes such as MAPK kinases related to cell wall integrity and genes involved in reactive oxygen species accumulation (Weiberg et al., 2013). This strategic employment of small RNAs enabled the pathogen to seize control of host immune responses, thereby facilitating successful invasion (Zhang et al., 2016a,b, 2022). The trans-kingdom miRNAs represent an innovative class of effector molecules adept at quelling host immune response. Notably, the miRNA (Bc-siR37) from *Bostalospora cinerea* was significantly enriched in the host *Arabidopsis* during the early stage of invasion. Bc-siR37 targeted a minimum of 15 genes, including WRKY transcription factors and cell wall modifying enzymes in *Arabidopsis* (Wang B. et al., 2017). Trans-kingdom miRNAs regulate host target genes to help pathogens successfully invade and colonize. Similarly, in the context of wheat stripe rust, Pst-mi1R1 was identified as a key pathogenic factor of the pathogen, which perturbs the host by targeting the gene *SM638* in the genome of the host wheat. Partial silencing of *SM638* in wheat significantly reduced the resistance to wheat stripe rust in resistant varieties (Wang M. et al., 2017). Recent results show have extended the scope of trans-kingdom RNA silencing to parasitic plants, exemplified by *Cuscuta campestris*. Endogenous miRNAs from this parasitic plant can be transported to the host *Arabidopsis thaliana*, guiding and modulating the parasitization process by regulating the expression of different target genes in the host (Hudzik et al., 2023). This study underscore the intricate involvement of miRNAs in the host immune pathway, shedding light on a previously uncharted aspect of parasitic interactions.

The trans-kingdom RNAi constitutes a pivotal mechanism in bolstering plant defenses against pathogens, exhibiting bidirectional transmission capabilities between host and pathogen. Trans-kingdom RNAi vectors were constructed using Host-Induced Gene Silencing (HIGS) to silence pathogen virulence-associated target genes, thereby allowing the host to acquire resistance against the pathogen. Guo et al. (2017, 2021) used HIGS technology to produce genetically stable transgenic cotton. Herein, the *VdH1* gene of *Verticillium dahlia*, pivotal for nuclear integrity, was effectively silenced, causing a reduction in virulence due to the loss of nucleus formation in the pathogen. This study is the first to demonstrate the potential of HIGS technology in plant disease resistance research (Zhang et al., 2016a,b). In a similar vein, Govindarajulu et al. (2015) harnessed HIGS technology to produce transgenic lettuce resistant to *Bremia lactucae*. By attenuating a key lettuce gene through RNAi, resistance against the pathogen was realized (Zhang et al., 2016a,b). Likewise, Chen et al. (2016) yielded transgenic wheat with enhanced resistance against *Fusarium graminearum* through HIGS-mediated silencing of the *FcGls1* gene, impairing mycelial cell wall integrity. These remarkable instances offer valuable insights for crop breeding via inter-species RNAi applications.

Evidently, trans-kingdom miRNAs exhibit a propensity for horizontal transfer across several species, such as from humans to malignant malaria-causing insects (*Plasmodium falciparum*) (LaMonte et al., 2012); from bacteria to insects (LaMonte et al., 2012); from parasitic plants to host plants (Shahid et al., 2018; Hudzik et al., 2023); from plant to insect (Ibrahim et al., 2011); from fungal pathogens to plants (Weiberg et al., 2013), from plant-to-fungal pathogens (Cai et al., 2018); and even reciprocal transfers between plants and insects (Wang et al., 2016). However, a notable gap exists in the exploration of transferable miRNAs in melon plants resistante to the parasitic broomrape. This research aims to unravel the intriguing implications of this novel defense strategy within the interaction between melon and broomrape. By delving into the dynamics of trans-kingdom RNAi, it aspires to shed light on the broader landscape of cross-border RNAi application in plant disease management. Furthermore, this research holds the potential to guide the evolution of pathogenic bacteria as miRNA targets, thereby advancing innovative strategies for combatting broomrape in melon cultivation.

## 2 Materials and methods

### 2.1 Plant materials and manipulations

**Figure 1:**
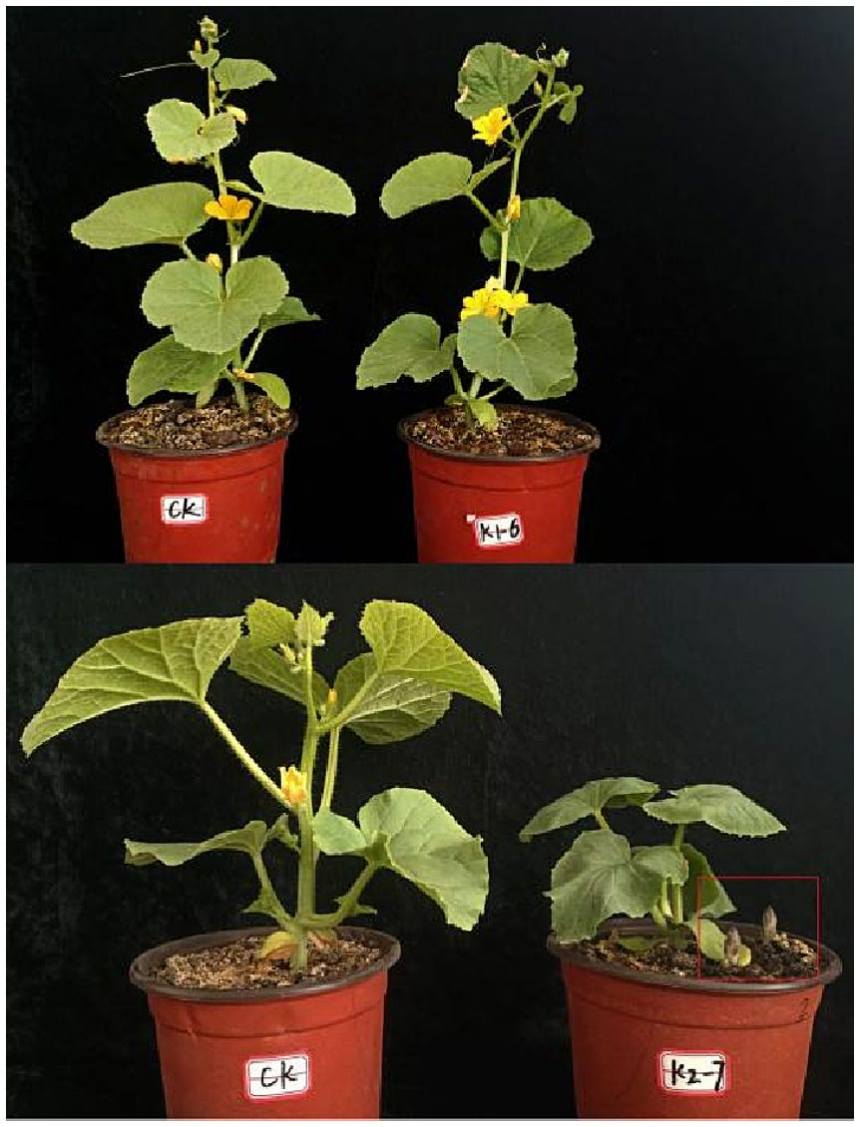
Resistant and susceptible lines were inoculated for 25 days. K1-6 is resistant line and K2-7 is susceptible line, the red box indicates the parasitic broomrape.

The resistant line K1-6 and sensitive line K2-7 melon cultivars are preserved in Xinjiang Academy of Agricultural Sciences (Figure 1). Broomrape seeds, identified as *Phelipanche aegyptiaca* Pers, were collected in the Kashgar region of Xinjiang. The pots (Diameter: 16 cm, Height: 12.5 cm) were filled with a vermiculite-sand mixture (1:1) and infused with 0.02 g of broomrape seeds. Each pot was planted with 1–2 melon seeds, and this setup was repeated 10 times for each treatment. The pots were placed randomly in a greenhouse (Temperature 28°C, light/darkness = 16: 8), receiving regular watering. Root samples from the parasitized melon were collected at 0 days (non-infectious treatment) and 25 days after infection for the resistant line K1-6 (R0 and R25) and sensitive line K2-7 (F0 and F25). Parasitic broomrape samples were taken for degradome sequencing to find target genes. Each sample was repeated 3 times, rapidly frozen in liquid nitrogen and stored at-80°C. The blank control was induced by strigolactone in Petri dishes and sampled after broomrape germination.

### 2.2 Construction of small RNA library

The broomrape samples were sent to Genepioneer Biotechnologies for small RNA sequencing. Total RNA was extracted using the RNA Easy Fast (DP452), and small RNA libraries were prepared using the TruSeq Small RNA Sample Prep Kits (Illumina, San Diego, United States) according to standard procedures. After library preparation, the constructed libraries were sequenced using Illumina Hiseq 2000/2500 with a sequence reading length of 1 × 50 bp.

### 2.3 miRNA sequencing and data processing

The initial sequencing data was aligned with the reference genome, with subsequent filtration to compare with the broomrape genomes. The remaining data were analyzed using the miRNA data analysis software ACGT101-miR (LC Sciences, Houston, Texas, United States). The analysis process of the software is as follows: elimination of 3′ junction and irrelevant sequences to get clean data; Length screening, filtering out data with baseline length at 18–25 nt in the clean data; assessment against various RNA databases (mRNA, RFam, Repbase) to exclude non-miRNA data, retaining valid sequences for further analysis.

### 2.4 miRNA identification

miRNA identification includes both known and novel miRNAs. We compared effective data with plant miRNA precursors and maturing sequences in the miRbase database. This comparison involved BLAST analysis of unique sequences spanning 18–25 nucleotides against a specific species precursor in miRBase (miRBase 21.0; http://www.mirbase.org/), in order to identify known miRNA as well as novel miRNA.

### 2.5 Degradome sequencing identifies cross-border miRNA target genes

#### 2.5.1 Destruction group building

The samples were sent to Genepioneer Biotechnologies for degradome sequencing. The proposed library steps are as follows: miRNA capture by magnetic beads, adaptor ligation; mixed reverse transcription of Biotinylated Random Primers and mRNA; PCR amplification, after completing the whole library preparation, the constructed library was sequenced with Illumina Hiseq 2000/2500, and the sequencing read length was 1 × 50 bp at the single end.

### 2.6 Degradome data analysis and target gene prediction

The raw data obtained from sequencing were processed through a series of data processing to obtain comparable sequenced pairs for subsequent analysis. The comparable pair sequences were compared with the melon cDNA database sequences to generate the degradome density files. The target gene mRNA sequences paired with the small RNA sequences of sequenced species were predicted by the Target finder target gene prediction software. The Allen Score was used to evaluate the degree of base complementarity between miRNA and target gene binding sites, indicating the binding site pairing penalty (where lower values indicate better pairing).

### 2.7 Target gene prediction

The target genes corresponding to melon miRNAs predicted by Target finder software based on the principle of base complementary pairing. The mRNAs in the generated degradome density files were combined and analyzed to identify the target genes of the miRNAs. Functional annotation of the target genes was performed by GO enrichment analysis and KEGG enrichment analysis.

### 2.8 qRT-PCR

Total RNA was digested by DNase I and cDNA were synthesized using Rrans Script Green miRNA Two-Step qRT-PCR SuperMix. Differential miRNAs were randomly chosen for validation, and primers were designed accordingly. Each sample underwent triplicate repetitions to determine relative expression of the target miRNA, H_2_O was used as a negative control and CmADP was used as a reference gene. The primers used in RT-PCR are listed in Supplementary Table S1.

## 3 Results

### 3.1 sRNA sequencing in melon varieties resistant or sensitive to broomrape

Here, using small RNA sequencing in melon root systems, we investigated miRNA expression variation in resistant and susceptible cultivars pre-and post-infection by the parasitic plant, broomrape (Figure 1). The results showed that a total of 12 melon sRNA libraries were prepared for sequencing on the Illumina SE50 platform. The integrity of the sequencing data was notably robust, with each of the four samples yielding a range of clean data reads (ReadSum) spanning from 11,023,148 to 11,977,704, and a clean date base (BaseSum) ranging from 562,180,582 to 610,862,904. These data were characterized by outstanding quality, as evidenced by Q20 > 98% and Q30 > 96% for each sample and GC content with a consistent range from 52.99 to 54.27% (Table 1). The above results indicate that the quality of the raw data obtained from sequencing meets the requirements for subsequent analysis.

**Table 1:** sRNA classification annotation statistics.

Type	F0	F25	R0	R25
rRNA	1,847,784 (40.7%)	2,563,363 (45.9%)	2,545,300 (49.6%)	2,357,108 (46.7%)
tRNA	252,608 (5.5%)	293,954 (5.2%)	288,017 (5.6%)	242,178 (4.9%)
snRNA	75 (0.0%)	115 (0.0%)	113 (0.0%)	83 (0.0%)
snoRNA	45,619 (1.1%)	50,316 (0.9%)	65,543 (1.3%)	39,611 (0.8%)
Repeat	1,586,690 (34.57%)	1,602,387 (28.6%)	1,380,110 (26.9%)	1,436,123 (28.6%)
miRNA	19,303 (0.4%)	7,219 (0.1%)	7,936 (0.2%)	2,285 (0.4%)
Other	811,715 (1.8%)	1,099,407 (19.2%)	842,163 (16.4%)	964,461 (10.1%)

**Figure 2:**
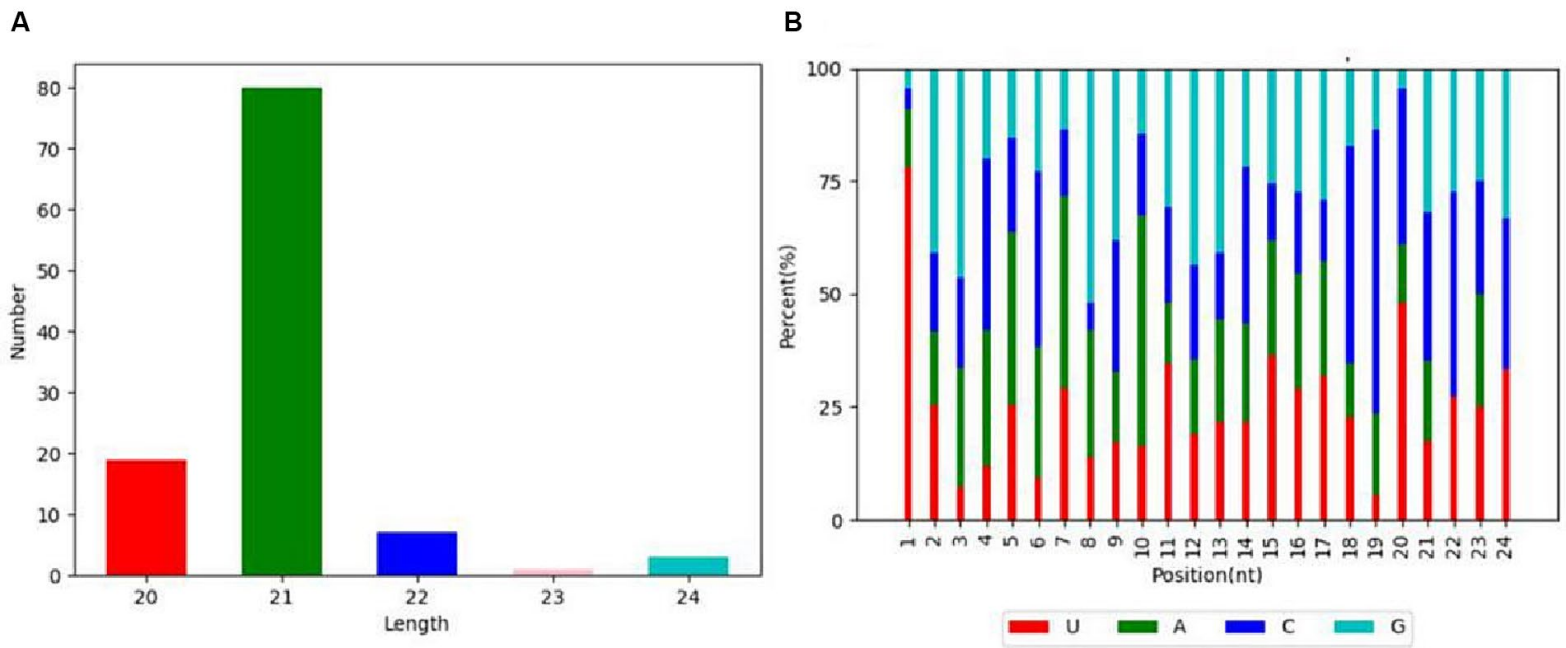
miRNA length distribution and nucleotide bias at each position. **(A)** The length distribution chart of identified miRNA. **(B)** Distribution of bases at each miRNA site.

The length distribution statistics were performed on the clean reads. The distribution of fragment lengths serves as an illuminating marker to identify small RNA species. miRNAs were usually concentrated at 21–22 nt, as shown in Figure 2A. The small RNA lengths of the samples were significantly concentrated at 21 nt. In addition, an insightful examination of the first point base preference in clean reads was conducted. A robust preference for the base pair U at the initial position was pronounced in the context of 21 nt fragments (Figure 2B). The results with the annotation of small RNAs are shown in Table 1.

### 3.2 miRNA identification

**Figure 3:**
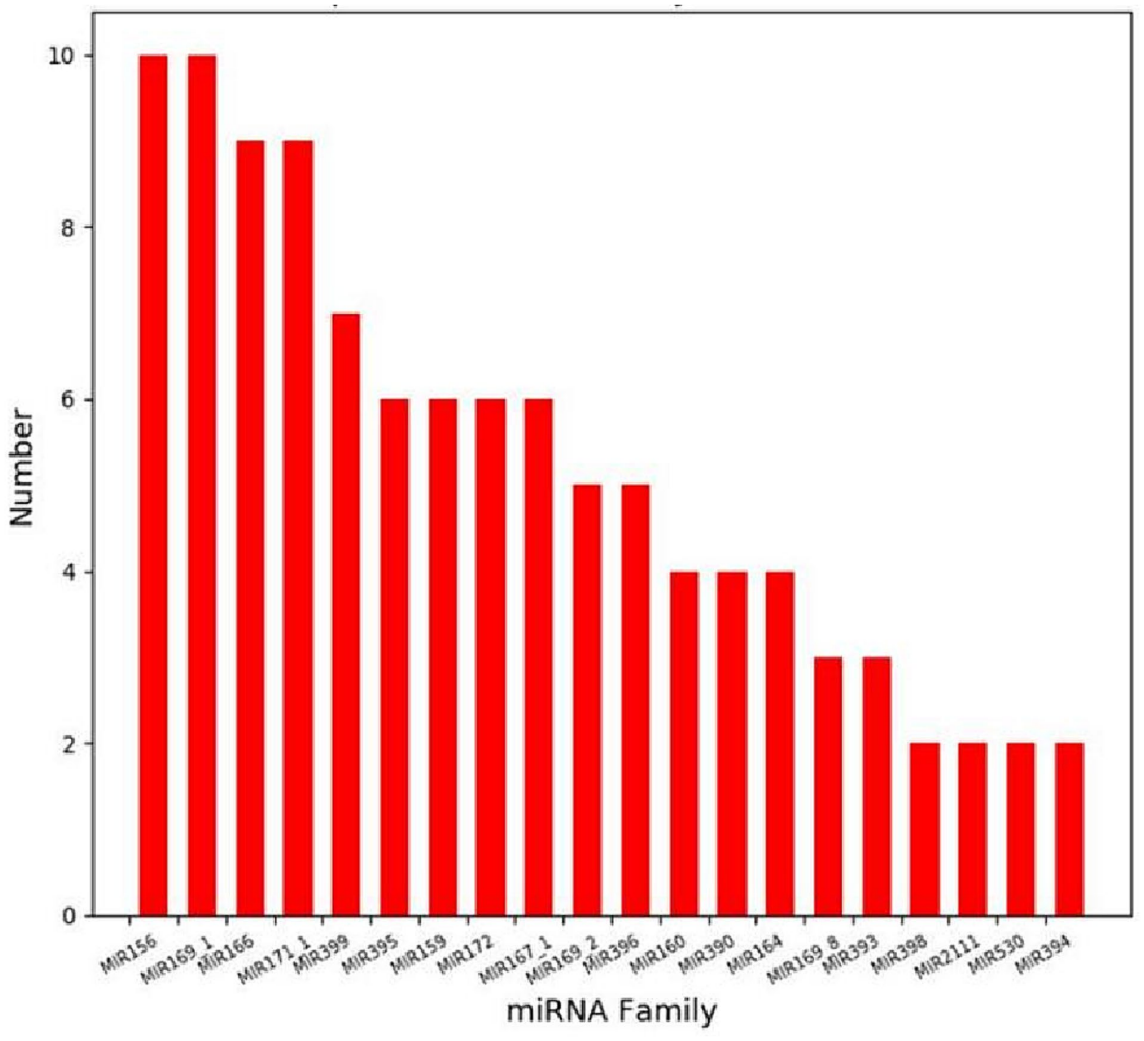
Top 20 miRNA family distribution.

The clean reads after removing the sequences of non-miRNAs were compared with plant miRNA precursors and mature sequences in the miRBase database. A total of 979 known miRNAs and 110 novel miRNAs were identified, which belong to 111 miRNA families. These miRNA families contain miRNA members ranging from 1–10. Notably, the MIR156 family emerged as the most populous (Figure 3), harboring an array of members such as cme-MIR156a, cme-MIR156b and cme-MIR156c. These miRNAs, closely linked with defense mechanisms and the accumulation of secondary metabolites in host plants, underscore their significance within this context.

### 3.3 miRNA differential expression analysis

**Figure 4:**
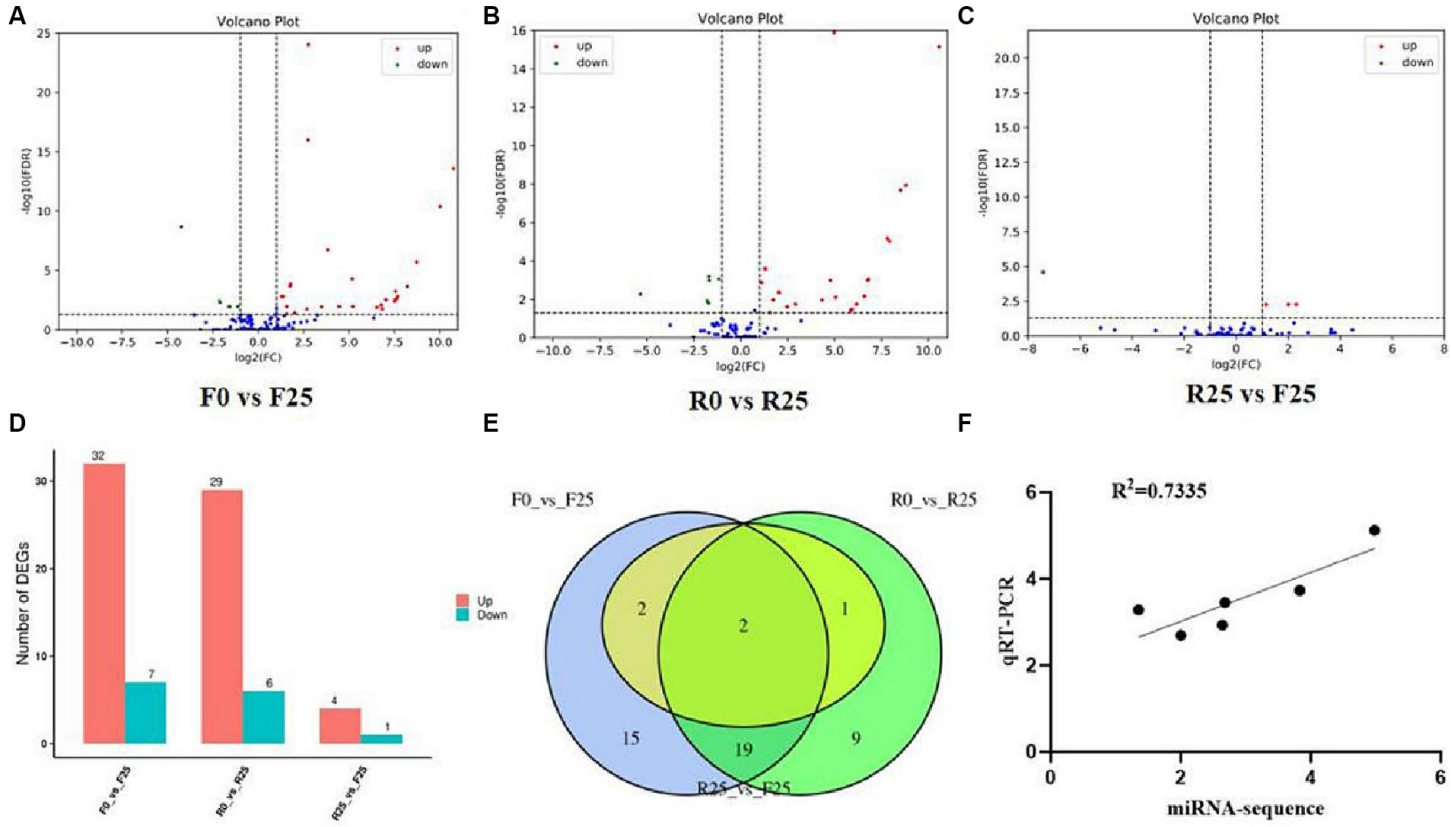
Differential expression of miRNA volcanic plot and Venn diagram. **(A)** miRNA volcano plot of sensitive line at 0 and 25 days after inoculation. **(B)** miRNA volcano plot of resistant line at 0 and 25 days after inoculation. **(C)** miRNA volcano plot of differences between sensitive and resistant lines after 25 days of inoculation. **(D)** Histogram of differential miRNA statistics. **(E)** Venn diagram of differentially expressed miRNAs. **(F)** Correlation analysis between qRT-PCR and miRNA sequencing results. The horizontal coordinate of the **A–C** plots indicates the change of miRNA expression ploidy in melon after being parasitized by broomrape; the vertical coordinate indicates the significance level of expression difference. The expression of up-regulated miRNA is represented by red dots, and the down-regulated miRNA by green dots indicates that the blue dots have not changed significantly.

Employing high-throughput sequencing, the systematic exploration of differential gene expression during biotic stress affords a rapid avenue for the annotation, analysis, and prediction of target genes, thereby facilitating a deeper comprehension of the intricate molecular mechanisms underpinning plant resistance to biotic stress. Our differential expression miRNA analysis for F0 and F25 revealed a total of 39 differentially expressed miRNAs, comprising 32 miRNAs significantly up-regulated and 7 significantly down-regulated (Figures 4A,D). For R0 and R25, we observed 35 differentially expressed miRNAs, with 29 displaying upregulated expression and 6 evincing downregulated expression (Figures 4B,D). A comparative analysis between R25 and F25 unveiled 5 differentially expressed miRNAs, comprising 4 up-regulated and 1 down-regulated (Figures 4C,D).

Furthermore, in order to elucidate the mechanism of resistance to Broomrape, this study focused on the differences in miRNA expression before and after broomrape infection in K1-6 (R25 VS R0) and in K2-7 (F25 VS F0). The results showed 22 miRNAs exhibiting consistent significant differential expression trends in R25 VS R0 and F25 VS F0, comprising 4 miRNAs down-regulated and 18 up-regulated in both comparisons. Interestingly, two miRNAs, namely cme-miR408 and cme-miR398a, displayed up-regulation across R25 VS R0, F25 VS F0 and R25 VS F25 comparisons (Figure 4E).

To verify the accuracy of miRNA sequencing data, a random selection of 6 miRNAs, including cme-miR408, cme-miR398a, cme-miR394a, cme-miR397, cme-miR477a and cme-miR160a, was subject to quantitative real-time PCR (qRT-PCR) analysis. The results showed a high degree of correlation between the qRT-PCR and sequencing data of the selected miRNAs, indicating that the miRNA sequencing data in this experiment were authentic and reliable (Figure 4F).

### 3.4 Degradome sequencing for identification of miRNA target genes

**Figure 5:**
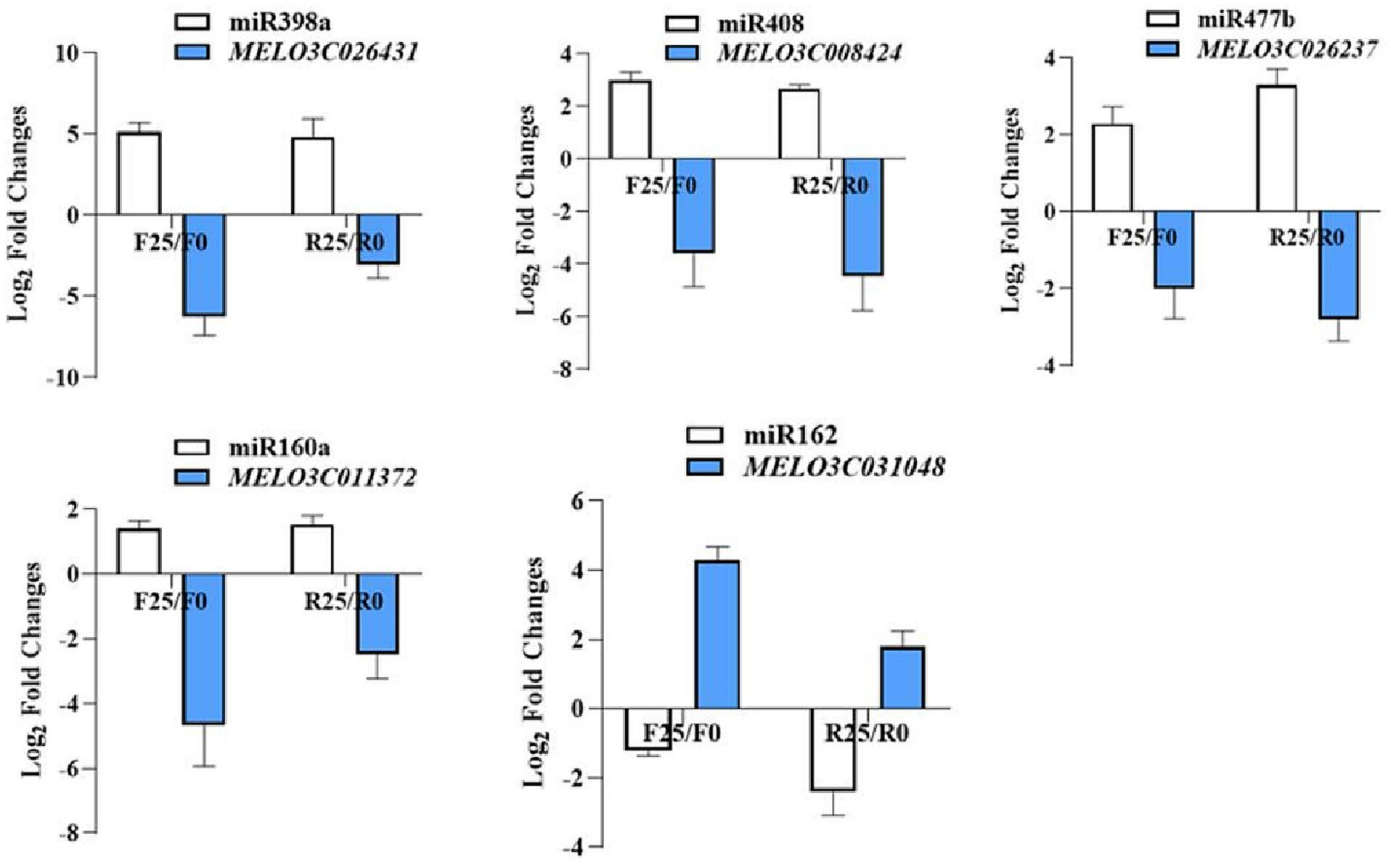
The verify of miRNA-target gene expression by qRT-PCR.

In order to gain deeper insights into the role of melon miRNAs in response to broomrape infestation, this study used degradome sequencing to identify the target genes of miRNAs. The degradome library was constructed from a mixed sample of F0, F25, R0 and R25. The data was compared to a quality control to predict the target genes of miRNAs. A total of 172 miRNA-targeted pairs were detected. In a bid to validate the predictive accuracy of these miRNA-target interactions, a judicious selection of 5 pairs of miRNA-targets for subsequent validation, including cme-miR398a-MELO3C026431, cme-miR408-MELO3C008424, cme-miR477b-MELO3C026237, cme-miR160a-MELO3C011372, and cme-miR162-MELO3C031048. The results showed a compelling negative correlation between the expression trends of miRNAs and their corresponding target genes, indicating that the miRNA target genes found by degradome were reliable (Figure 5).

### 3.5 Functional enrichment of differential miRNA target genes

**Figure 6:**
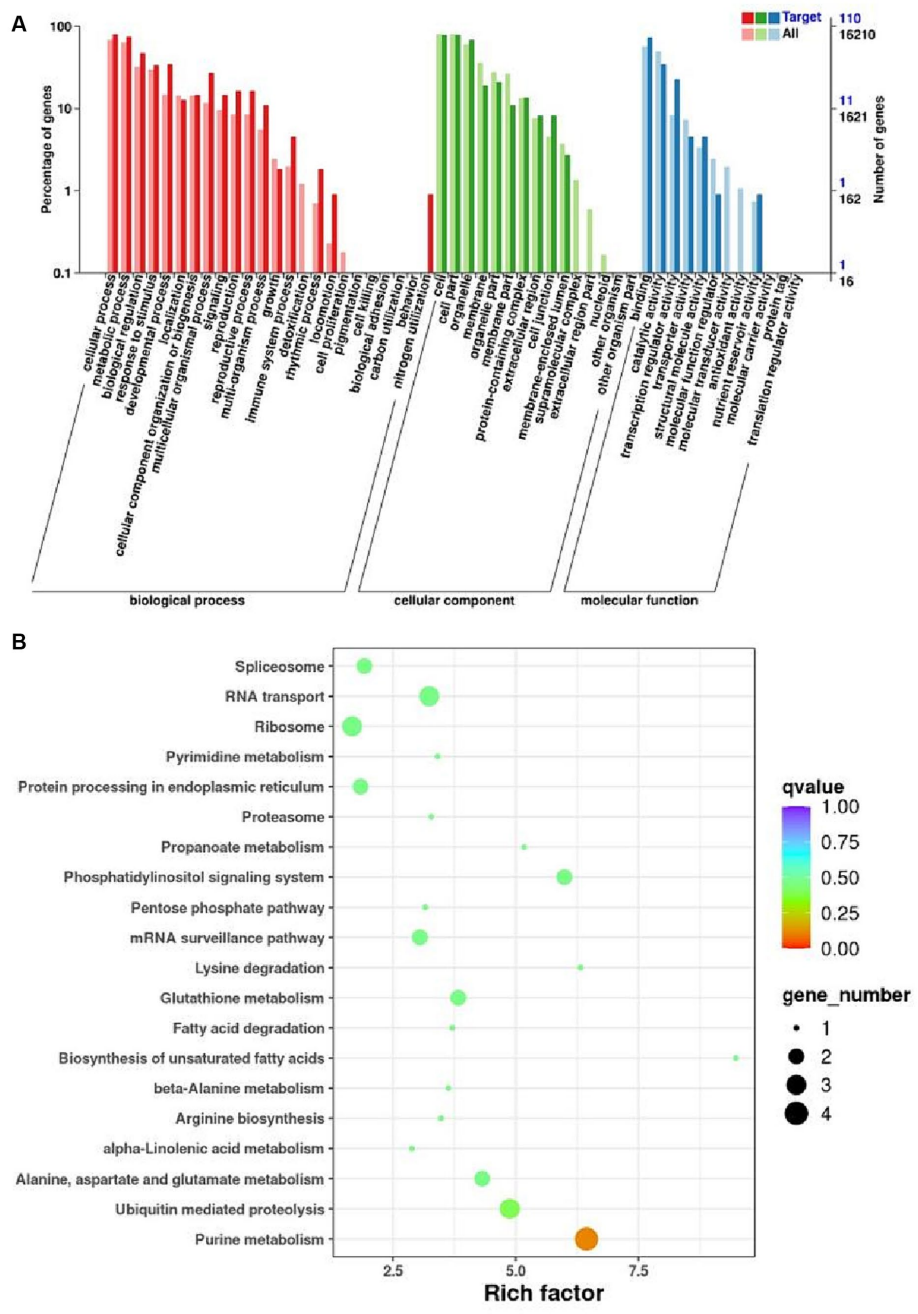
Functional annotation of the target genes of the differential expressed miRNAs. **(A)** GO enrichment analysis of differentially expressed miRNA target genes. The horizontal coordinate is the GO function entry and the vertical coordinate is the number of differential genes. **(B)** Analysis of KEEG enrichment pathway for differentially expressed miRNA target genes. Each row in the graph represents a KEGG pathway. The horizontal coordinate is the enrichment factor.

The miRNA target genes identified by degradome sequencing were subject to GO enrichment analysis. The corresponding GO annotations were classified into three categories according to biological process, cellular component and molecular functions, enriching 432, 341, and 156 target genes, respectively. The target genes were significantly enriched in cellular and metabolic processes in the biological process category. In addition, the target genes were significantly enriched in cells, cell parts and binding within the cellular component and molecular functions domain (Figure 6A).

To glean further insights into the intricate pathways associated with melon miRNAs’ response to broomrape infestation, a comparative analysis was pursued by aligning the miRNA-target genes with the KEGG database. Target genes are enriched in phosphatidylinositol signaling and purine metabolism, which are important pathways involved in the defense response of melon. The results underscore the importance of miRNA regulation in orchestrating melon’s defense response to broomrape infestation (Figure 6B).

### 3.6 Transferable miRNA screening

We identified a total of four known miRNAs transferred from melon to broomrape through screening of the transferable miRNA, which were cme-miR156a, cme-m0671-5p, cme-m0738-3p, and cme-miR398a. These miRNAs are mainly involved in structural constituent of cell wall, transposon protein, glutamate receptor and cysteine-rich repeat secretory protein (Table 2). From the functions of these transferred miRNAs, it is speculated that the miRNAs transferred from melon to broomrape may inhibit lepidote growth by affecting the biosynthesis of lepidote miRNAs.

**Table 2:** Transferable miRNA and target gene annotation information.

Micro RNA	Target gene ID	Gene position		CDs (bp)	Target gene annotation	Arabidopsis homology
		Start	End (+/−)			
cme-miR156a	MELO3C019024	15,870,317	15,872,012 (−)	1,146	At5g64090	AT5G64090.1
cme-m0671-5p	MELO3C035153	26,029,432	26,037,682 (−)	1,156	CACTA En-Spm transposon protein	AT3G56600.3
cme-m0738-3p	MELO3C010853	28,110,891	28,112,794 (−)	1,668	Glutamate receptor 2.5-like	AT5G11210.9
cme-miR398a	MELO3C026431	21,484,312	21,487,759 (−)	906	Cysteine-rich repeat secretory 12-like protein	AT2G01660.3

## 4 Discussion

miRNAs represent a class of non-coding RNAs pivotal for regulatory functions that exert critical influences in almost all aspects of plant growth and development. Additionally, they play important regulatory roles in response to environmental variables such as light and nutrients, and both biotic and abiotic stresses (Budak et al., 2015; Shriram et al., 2016; Aslam et al., 2022; Kajla et al., 2023). The advent of small RNA sequencing, based on high-throughput sequencing, has effectively dismantled barriers in miRNA research, facilitating a more incisive exploration of their biological functions. While some reports have used small RNA sequencing to study melon miRNAs, the context of broomrape resistance in melon remains elusive. In this study, a total of 979 known miRNAs and 110 novel miRNAs were identified, belonging to 111 miRNA families. Intriguingly, the MIR156 family emerged as the most prolific, associated with plant resistance and accumulation of secondary metabolites (Cui et al., 2014; Wang, 2014; Rahimi et al., 2022). The results imply a potentially pivotal role for this miRNA family in the resistance of melon to broomrape infestation.

In this study, small RNA sequencing was performed to identify and quantify the expression of miRNAs in the root systems of susceptible and resistant melon lines before and after being infected by broomrape. The differential expression profiles were constructed by comparing the miRNA expression of resistant and susceptible strains. The Venn diagrams unveiled noteworthy distinctions, with cme-miR398a and cme-miR408 standing out as significantly divergent across all examined combinations. It is worth stating that while we identified the differentially expressed miRNAs in resistant and susceptible lines by small RNA sequencing, the target genes of these miRNAs and their regulatory functions remain unknown, which can be well addressed by degradome sequencing. In comparison, predictive target genes based on sequence complimentarity often entail high rates of false positives. Alternately, experimental approaches, though comprehensive, suffer from limitations of expense and time. In this context, degradome sequencing presents an elegant solution. The degradome sequencing technology is based on the principle that plant miRNAs can shear target mRNAs. The intersection of the sheared mRNA fragments are collected and sequenced by high-throughput sequencing technology, thereby allowing the prediction of miRNA target genes by Target finder software. The degradome sequencing effectively overcome the drawbacks and defects of the previous methods for identifying miRNA targets. The target genes of these two miRNAs encode cysteine-rich repeat secretory protein and copper-transporting ATPase. A recent study showed that both proteins are associated with the defense response of the organisms (Tao et al., 2021). Therefore, we speculate that these two miRNAs and their target genes play an important role in the resistance of melon to broomrape infestation.

It is well documented that there is a complex transfer of substances between the host and the parasitic organism, including miRNA, and that these substances have essential effects on both parties after the transfer. miRNAs can be transferred through the host to the parasitic plant and improves the host resistance by RNA interference. It has been shown that *Cuscuta campestris* transfers a large number of miRNAs to the host Arabidopsis and Benthamiana, which may function as virulence factors during parasitization as cross-species regulators of host gene expression. However, the extent of long-distance miRNA movement during broomrape parasitization has remained a question. The results of this study show that certain miRNAs are transferred from resistant melon varieties to broomrape. One of the screened transfer miRNAs, cme-miR156a, is a very conserved small non-coding RNAs in plants, and its target gene family is the SPL (SQUAOSA promoter binding like) family (Matthew et al., 2002; Bonnet et al., 2010). The miR156-SPL pathway plays a regulatory role in several plant growth and developmental processes, including phytohormone signaling and stress response (Wang, 2014; Lei et al., 2016; Miao et al., 2019). Therefore, it is hypothesized that the melon can initiate defense response through the overexpression and transfer of miR156 to broomrape, thus affecting the infestation process of broomrape. In addition, miR398a is mainly involved in the negative regulation of dismutase genes (Raichur et al., 2007; Zidenga et al., 2012). When melon was infected by broomrape, the expression of cme-miR398a in the roots was elevated, indicating that cme-miR398a may increase ROS content by inhibiting the activity of reactive oxygen species (ROS) enzymes in the cells at the infected site, which eventually resulted in an allergic necrotic response and prevented broomrape infestation.

In this study, a combination of small RNA sequencing and degradome sequencing was used to identify the differentially expressed miRNAs and their target genes between susceptible and resistant lines when infested by broomrape and to explore the defense response of melon against broomrape. In addition, 4 transferable miRNAs were identified in the analysis of melon and broomrape interactions. This comprehensive investigation into the novel defense strategy that underpins melon’s interaction with broomrape furnishes valuable reference insights, with implications extending to the broader application of cross-border RNA interference in the realm of plant disease control.

## Data availability statement

The datasets presented in this study can be found in online repositories. The names of the repository/repositories and accession number(s) can be found in the article/Supplementary material.

## Author contributions

J-CM: Writing – original draft, Writing – review & editing. MY: Writing – review & editing. J-HL: Funding acquisition, Writing – review & editing. J-YY: Writing – original draft. H-JW: Funding acquisition, Writing – original draft, Writing – review & editing.
