# Supplementary material for: The role of small RNAs in resistant melon cultivar against Phelipanche aegyptiaca parasitization
Source: Front Microbiol. 2024 May 7;15:1408926. doi: 10.3389/fmicb.2024.1408926 (PMC11106454; doi:10.3389/fmicb.2024.1408926)
Supplement: Supplementary file 1 [file Table_1.DOCX]

Supplementary Material

The Role of Small RNAs in Resistant Melon Cultivar against *Phelipanche aegyptiaca* Parasitization

# Supplementary Tables

**Supplementary Table 1** qRT-PCR primers

| Primer names | Primer sequence( 5^,^-3^,^) |
| --- | --- |
| cme-miR408 | AUGCACUGCCUCUUCCCUGGC |
| cme-miR398a | UGUGUUCUCAGGUCGCCCCUG |
| cme-miR394a | UUGGCAUUCUGUCCACCUCC |
| cme-miR397 | UCAUUGAGUGCAGCGUUGAUG |
| cme-miR477a | ACCUCCCUCAAAGGCUUCCAA |
| cme-miR160a | UGCCUGGCUCCCUGUAUGCCA |
| cme-miR477b | CUCUCCCUCAAAGGCUUCUG |
| cme-miR162 | UCGAUAAACCUCUGCAUCCAG |
| MELO3C026431-F | TCTCTTCCGTATCATCTCTCT |
| MELO3C026431-R | GTGAGAAGGGAGTTGAGA |
| MELO3C008424-F | GCAAAATGGGGAATTAAGGTG |
| MELO3C008424-R | GTTTTGCAGGAGGCGTACCCA |
| MELO3C026237-F | CACGATGGTGAAGCTTTGGTCA |
| MELO3C026237-R | ATTTCGGCAACATGGCATCCA |
| MELO3C011372-F | CTCATCATCCCCAAATTCTCTC |
| MELO3C011372-R | GAACCGGCGGTGATCGAACAA |
| MELO3C031048-F | TGGGGAGATTGGAGCAGTGAT |
| MELO3C031048-R | CCGACAAATAAGACTTGTTGG |
| CmADP | F:ATATTGCCAACAAGGCGTAGA |
|  | R:TGCCCGTAAACAAGGGATAAA |

**Supplementary Table 2** Raw data statistics

| Sample | ReadSum | BaseSum | Cycle Q20(%) | Q20(%) | Q30(%) | GC(%) |
| --- | --- | --- | --- | --- | --- | --- |
| F0 | 11023148 | 562180582 | 100 | 98.86 | 96.47 | 54.27 |
| F25 | 11866804 | 605207021 | 100 | 98.90 | 96.49 | 53.53 |
| R0 | 11913852 | 607606469 | 100 | 98.80 | 96.23 | 52.99 |
| R25 | 11977704 | 610862904 | 100 | 98.70 | 95.97 | 53.96 |

**Supplementary Table 3** Summary of small RNA sequencing data from four samples

| Read types | F0 | F25 | R0 | R25 |
| --- | --- | --- | --- | --- |
| Total reads | 11023149 | 11866804 | 11913852 | 11977704 |
| Reads with adapters | 10889045(98.8%) | 11828831(99.7%) | 11845492(99.4%) | 11842921(98.9%) |
| ＜18nt | 5889417(53.4%) | 5807291(48.9%) | 6276275(52.7%) | 6433165(53.7%) |
| ＞30nt | 569937(5.2%) | 442751(3.7%) | 508396(4.3%) | 502689(4.2%) |
| Clean reads | 4261430(38.7%) | 5616761(47.3%) | 5129181(43.1%) | 5041850(42.1%) |
| Total bases | 562180582 | 605207021 | 607606469 | 610862904 |

**Supplementary Table 4** miRNA detection results statistics

| **miRNA** | **Number** |
| --- | --- |
| Total | 1089 |
| Conservative miRNA | 110 |
| Unconservative miRNA | 979 |
